# Identification of QTL underlying the main stem related traits in a doubled haploid barley population

**DOI:** 10.3389/fpls.2022.1063988

**Published:** 2022-12-01

**Authors:** Anyong Zhang, Ting Zhao, Xue Hu, Yu Zhou, Yue An, Haiyi Pei, Dongfa Sun, Genlou Sun, Chengdao Li, Xifeng Ren

**Affiliations:** ^1^ College of Plant Science and Technology, Huazhong Agricultural University, Wuhan, China; ^2^ Hubei Hongshan Laboratory, Wuhan, China; ^3^ Department of Biology, Saint Mary’s University, Halifax, NS, Canada; ^4^ College of Science, Health, Engineering and Education, Murdoch University, Murdoch, WA, Australia

**Keywords:** barley (*Hordeum vulgare* L.), internode length, stem diameter, lodging resistance, quantitative trait loci (QTL)

## Abstract

Lodging reduces grain yield in cereal crops. The height, diameter and strength of stem are crucial for lodging resistance, grain yield, and photosynthate transport in barley. Understanding the genetic basis of stem benefits barley breeding. Here, we evaluated 13 stem related traits after 28 days of heading in a barley DH population in two consecutive years. Significant phenotypic correlations between lodging index (LI) and other stem traits were observed. Three mapping methods using the experimental data and the BLUP data, detected 27 stable and major QTLs, and 22 QTL clustered regions. Many QTLs were consistent with previously reported traits for grain filling rate, internodes, panicle and lodging resistance. Further, candidate genes were predicted for stable and major QTLs and were associated with plant development and adverse stress in the transition from vegetative stage to reproductive stage. This study provided potential genetic basis and new information for exploring barley stem morphology, and laid a foundation for map-based cloning and further fine mapping of these QTLs.

## Introduction

Lodging largely impairs grain yield and quality, especially for high-yielding cultivars (Islam et al., 2007), which is divided into stem lodging or root lodging (Kashiwagi et al., 2005). In cereal crops, lodging is governed by genetic, field management and environmental factors (Wu and Ma, 2016). In many agricultural systems, this is a tough challenge due to the complexity of stem lodging (Berry et al., 2004). Especially the large panicles of modern varieties are more prone to lodging in the presence of wind, rain and diseases (Martinez-Vazquez, 2016). Lodging affects photosynthetic capacity of cereal crops and has an adverse effect on grain development (Shah et al., 2017; Shah et al., 2019). Besides, lodged barley is likely to be infected with diseases and pests (Berry et al., 2004; Caier̄ao, 2006).

Crop yield is affected by the source, sink and flow. Stems are the most important storage organs during assimilation translocation after anthesis (Matthias et al., 2016), and plants particularly rely on the dry matter and nitrogen fixation of stems under stress (Housley and Peterson, 1982). As a regular conduit for transporting water and nutrients from root or leaves to panicle, stem structure is the key to improve grain filling and high yield (Chen et al., 2004; Huang et al., 2016; Zhai et al., 2018). Additionally, stem is the supporting organ for proper distribution of leaves, which is beneficial to improve the efficiency of sunlight use. Changes at the horizontal location of plants in photosynthesis can lead to 27−31% reduction in the yield (Pinthus, 1974; Berry et al., 2004). After heading of barley, the flag leaf is the main photosynthetic organ (Yap and Harvey, 1972), therefore, as a bridge from flag leaf to panicle, the uppermost internode is especially crucial in the later stage for filling of grain. Selection of lodging resistant cultivars is one of several very important targets in cereal breeding program. The lodging resistance correlated with stem strength, plant height, panicle type and cell wall components (Shah et al., 2017; Khobra et al., 2019). The dwarf and semi-dwarf genes were preferentially used to lower lodging risk in barley during the Green Revolution (Hedden, 2003), but several studies have shown that plant over short affected crop yield (Islam et al., 2007), so enhancing the stem strength of plants is another viable option to avoid lodging.

Many studies have been done on barley lodging (Cui and Shen, 2011; Baker et al., 2014; Leblicq et al., 2016). Based on stalk characteristics and lodging factors, a lodging index was proposed to measure the lodging trait (Islam et al., 2007; Li et al., 2017), thus, the lodging index becomes a comprehensive trait composed of many single traits. Moreover, some studies have indicated that leaf sheaths and vascular bundles are also crucial contributing factors to lodging (Wu and Ma, 2020; Cornwall et al., 2021). Lin et al. (2005) indicated that a lower pith diameter/stem diameter ratio can improve stem strength. The lignin and cellulose content of secondary cell wall (CW) also affects the mechanical properties of stems (Jones et al., 2001; McFarlane et al., 2014).

In recent years, many studies on stem related traits in barley were reported. Such as Bellucci et al. (2017) performed genome-wide association mapping of grain yield and cell wall polymer content in winter barley. QTLs for the length of each internode (Schmalenbach and Pillen, 2009), chromosome regions containing significant associations with cellulose concentration (Houston et al., 2015), characterization of plant height (Wendt et al., 2016; Bélanger et al., 2018; Pu et al., 2021) have been reported. Kristensen et al. (2016) identified some barley lodging resistance loci. The focus of lodging in barley has been on root, plant height and spike traits, limited research was performed on the effect of stem strength, stem internode and node diameter on lodging.

In the study, we performed QTL mapping of 13 stem related traits after 28 days of heading using a barley DH population, including uppermost node diameter (UND), second node diameter (SND), third node diameter (TND), uppermost internode length (UIL), second internode diameter (SID), third internode diameter (TID), second internode length (SIL), third internode length (TIL), uppermost internode diameter (UID), main stem length of fracture (MSL), stem fresh weight (SW), third internode breaking force (TIBF) and lodging index (LI). The aims were to explore the relationship between stem internode traits and lodging traits, and to predict and screen out candidate genes related to stem development. The study will deepen our understanding of the genetic basis of stem, and provide new insights to boost lodging resistance breeding in barley.

## Materials and methods

### Plant material

A doubled haploid (DH) population with 122 lines derived from six-rowed barley Huaai11 and two-rowed barley Huadamai 6 was employed to identify QTLs (Ren et al., 2010). These accessions were evaluated over two crop seasons (2020-2021, 2021-2022) in the experimental farm of Huazhong Agricultural University, Wuhan, China (30°C 48’N, 114°C 36’E), with three replicates in a completely randomized block design. In each replicate, lines were planted in double rows with 15 cm plant spacing and 20 cm row spacing. Field cultivation management followed standard agricultural practices for barley production.

### Phenotyping

The heading date of each line was recorded, after 28 days of heading, three plants with uniform growth were randomly selected from each replicate and their main stems were cut at the root to measure phenotypic data of stem related traits, and the mean value for each phenotype was used for the analysis. The main stem length (from the fracture of the stem to the apex of the panicle, MSL, cm), uppermost internode length (UIL, cm), second internode length (SIL, cm) and third internode length (TIL, cm) were measured using a straight edge, the uppermost node diameter (UND, mm), second node diameter (SND, mm), third node diameter (TND, mm), uppermost internode diameter (UID, mm), second internode diameter (SID, mm) and third internode diameter (TID, mm) were measured at the middle of them with a slide caliper. The breaking force of third internode (TIBF, N) was measured using a prostrate tester (DIK7400, Japan), and the fracture site was arranged in the center of the third internode (**Supplementary Figure S1**). Main stem fresh weight (SW, g) is the weight from the fracture to the apex of the panicle. Lodging index (LI) was calculated according to the equation: (Li et al., 2017)


$$\text{LI=}\frac{\text{MSL}\times \text{SW}}{TIBF/9.8\times 5\times 1000}\times 100$$

### Data analysis

The data was analyzed using SPSS 25 (USA). The best linear unbiased predictor (BLUP) of stem traits was forecast using the R package lme4. The broad-sense heritability (H^2^) was computed using: H^2^ =V_G_/(V_E_/N + V_G_), N was the number of environments (Liu et al., 2020). In this study, the BLUP was used to analyze the Pearson correlation between stem traits. The plots were drawn using R package ggplot2.

### QTL analysis

A high-density genetic linkage map of this double haploid population was constructed by Ren et al. (2016), which included 1962 markers on all seven chromosomes. It spanned 1375.80 cM of the whole-genome with an average marker distance of 0.7 cM. Stem related traits QTLs detection were analyzed in QTL IciMapping v4.1 software. The mapping method ICIM-ADD in “MET” module and “BIP” module was used to perform Multi Environment and Single environment Trials analyses, respectively. The PIN was 0.001 and the step was 1.0 cM. Furthermore, QTL analysis for BLUP was performed in BIP module. In addition, to overcome the interference of row type (Rt), we used Rt as covariates. QTL analysis of covariates was performed using QTL.gCIMapping software of R (Feng et al., 2018). The LOD was set to 3.0, and the step was 1 cM.

### Gene annotation for major QTLs

For the same trait, a QTL with an explained phenotypic variance (PVE) ≥10% and positioned in at least two years (including BLUP) as a stable QTL. To determine whether the QTL is novel, we compared the physical locations of loci detected here with those reported. The sequences of flanking markers were searched at the National Center for Biotechnology Information (NCBI). By searching the coding sequences, the predicted candidate genes of the main QTL in the physical interval were obtained from Barley genome assembly Morex_v2.0. The orthologs of other plants genes in the barley reference genome were identified using the Ensembl Plant Database (http://plants.ensembl.org/Hordeum_vulgare/Tools/Blast) (Mascher et al., 2017). We reviewed annotated information of the markers and identified potential candidate genes (Cantalapiedra et al., 2015).

## Results

### Phenotype analyses

**Figure 1:**
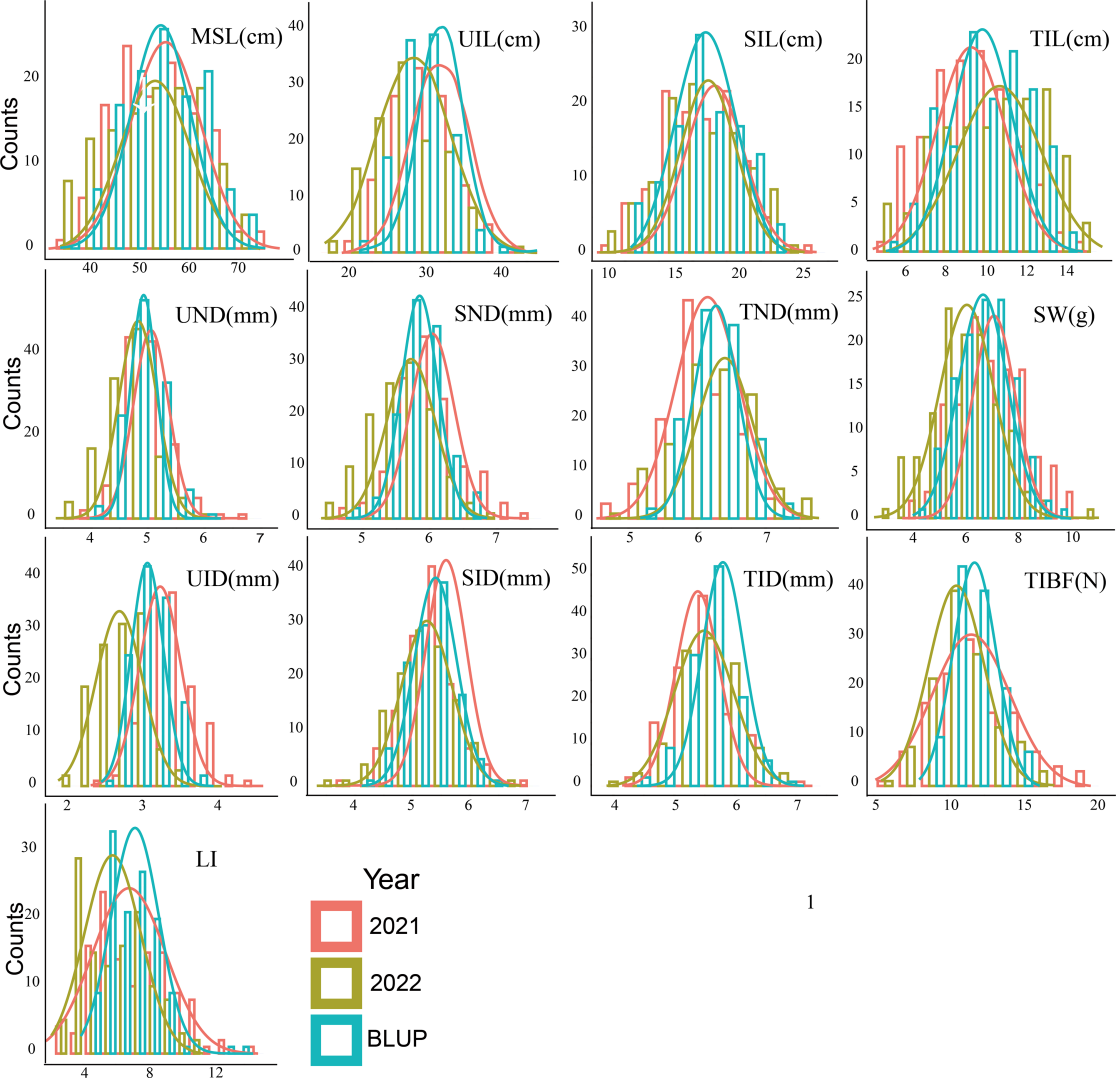
Phenotype distribution of 13 stem traits in each season (2021 and 2022) and BLUP value (best linear unbiased prediction environments).

We phenotyped 13 stem related traits 28 days after the heading in 2021 and 2022 (**Table 1**). Large variation of the stem traits was also found, the coefficient of variation (CV) was 8.09 -34.11%. Broad-sense heritability of these traits was 67.00-97.29%. The phenotypic differences in the stem of parents were shown in **Table 1**. T-tests showed significant differences (P< 0.05) in all stem related traits except TIBF and UND between parents. Huadamai 6 owned higher values for UND, SND, TND, SID, TID, UIL, SIL, TIL, MSL, SW, TIBF and LI in two years than Huaai11, while Huaai 11 had more UID than Huadamai 6. All 13 stem related traits showed normal distribution (**Figure 1**).

**Table 1:** Phenotypic performance for the thirteen stem related traits in the DH population and their parents.

Traits	Year	Huadamai6	Huaai11	T^a^	DH Lines						
		Mean	Mean		Mean	Min^b^	Max^c^	SD^d^	CV^e^	Corr^f^	H^2g^
UND (mm)	2021	5.49	4.81	**	5.10	3.89	6.66	0.43	8.43	0.76	90.08
	2022	4.92	4.72		4.61	3.47	5.95	0.44	9.54		
SND (mm)	2021	6.75	5.96	**	6.06	4.70	7.53	0.49	8.09	0.70	88.67
	2022	5.95	5.62	**	5.56	4.35	6.77	0.47	8.45		
TND (mm)	2021	6.67	6.06	*	6.09	4.11	7.49	0.53	8.7	0.64	91.12
	2022	6.90	6.47	*	6.26	4.99	7.70	0.57	9.11		
UIL (cm)	2021	40.22	20.51	**	27.24	18.45	40.22	4.25	15.6	0.74	92.78
	2022	37.78	19.15	**	24.64	15.20	37.70	4.55	18.47		
SIL (cm)	2021	19.87	12.28	**	15.81	9.54	23.71	2.95	18.66	0.85	97.05
	2022	16.04	11.61	**	15.72	8.49	22.39	3.05	19.4		
TIL (cm)	2021	13.07	7.82	**	9.48	5.42	14.81	2.41	25.42	0.85	67.00
	2022	11.90	6.14	**	10.26	6.89	15.25	2.76	26.9		
UID (mm)	2021	3.45	3.67	*	3.48	2.58	4.50	0.33	9.48	0.68	80.42
	2022	2.75	3.03	*	2.72	2.07	3.60	0.32	11.76		
SID (mm)	2021	5.97	5.39	**	5.36	3.96	6.90	0.47	8.77	0.75	93.92
	2022	6.09	5.56	*	5.17	3.39	6.83	0.55	10.64		
TID (mm)	2021	6.27	5.79	**	5.55	4.21	7.29	0.51	9.19	0.76	95.48
	2022	6.90	6.47	**	5.55	4.09	6.80	0.52	9.37		
LI	2021	10.79	5.09	**	7.27	2.70	14.61	2.42	33.29	0.80	84.41
	2022	9.94	4.86	**	5.98	2.75	10.85	2.04	34.11		
SW (g)	2021	8.51	6.82	*	7.22	3.98	10.32	1.33	18.42	0.69	90.56
	2022	8.10	6.77	*	6.04	2.96	10.94	1.29	21.36		
TIBF (N)	2021	12.40	11.60		12.14	6.61	19.33	2.80	23.06	0.87	84.32
	2022	12.53	11.17		11.11	6.45	16.68	2.12	19.08		
MSL (cm)	2021	78.04	43.60	**	55.44	37.79	78.04	8.71	15.71	0.86	97.29
	2022	72.46	40.03	**	53.89	35.00	74.20	9.84	18.26		

^a^T,: Student’s test, (*, p < 0.05; **, p < 0.01); ^b^Min, Minimum; ^c^Max, Maximum; ^d^SD, standard deviation; ^e^CV, coefficient of variation of a trait; ^f^Corr, correlation coefficient of two-year phenotypic data; ^g^H2, broad-sense heritability.

### Correlations between traits

**Figure 2:**
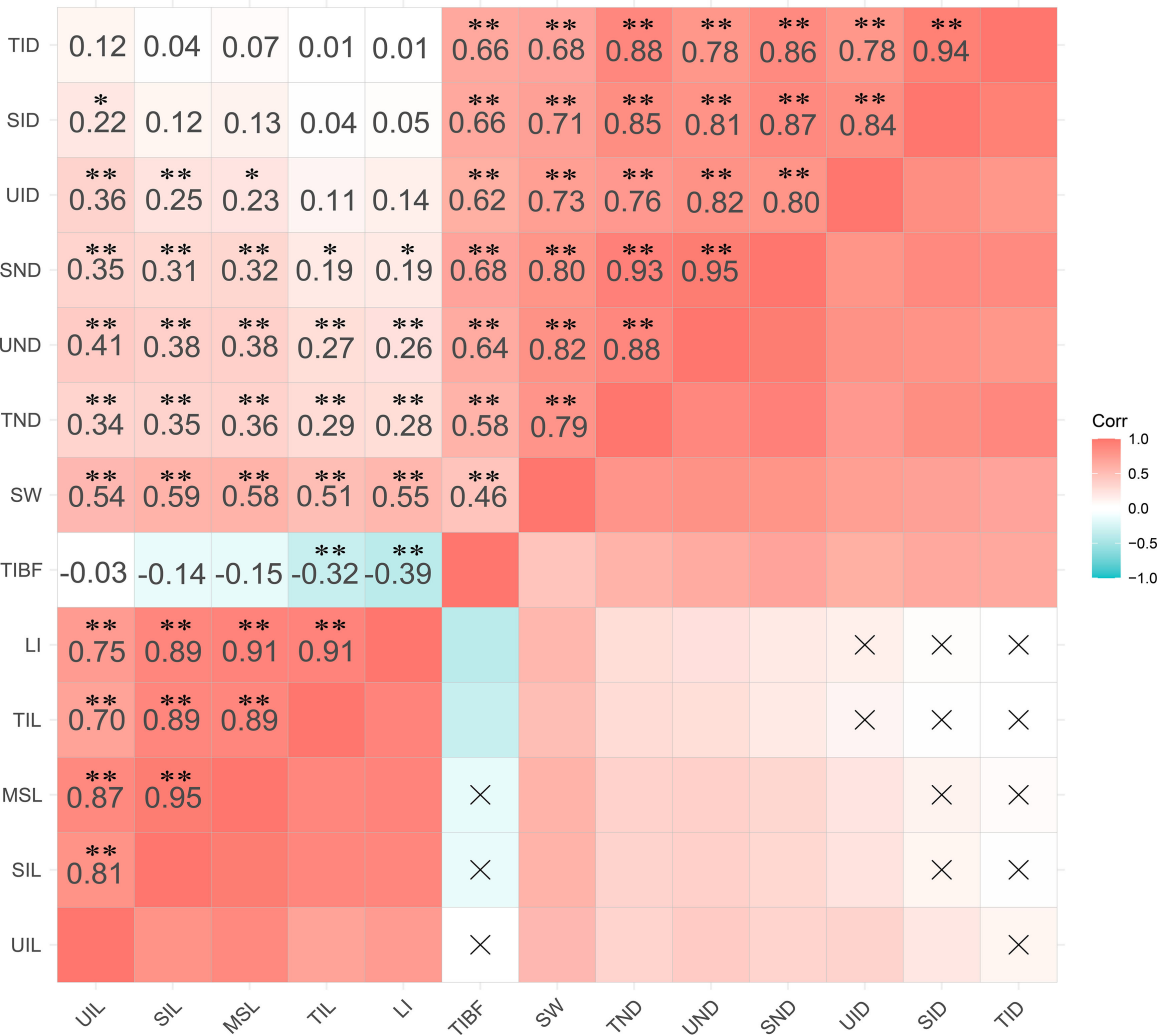
Pearson correlation coefficients among 13 stem traits of BLUP. The two-tailed t-test was applied to test the significance of correlation coefficients (*p < 0.05; **p < 0.01).

The best linear unbiased predictor (BLUP) of stem lodging traits was used for Pearson correlation analysis (**Figure 2**). The results showed that TIBF was negatively correlated with LI and TIL (P<0.01). UIL, SIL and MSL showed a significant correlation with UND, SND and TND (P<0.01), and the length of each internode had no correlation with TID. SID was positively correlated (P<0.5) with UIL. The diameter of each stem node was positively correlated with the stem diameter of each internode (P<0.01). SW was positively correlated with UIL, SIL, MSL, TIL, LI and TIBF. TIL and MSL had the highest correlation with lodging index (LI).

### QTL analysis

**Figure 3:**
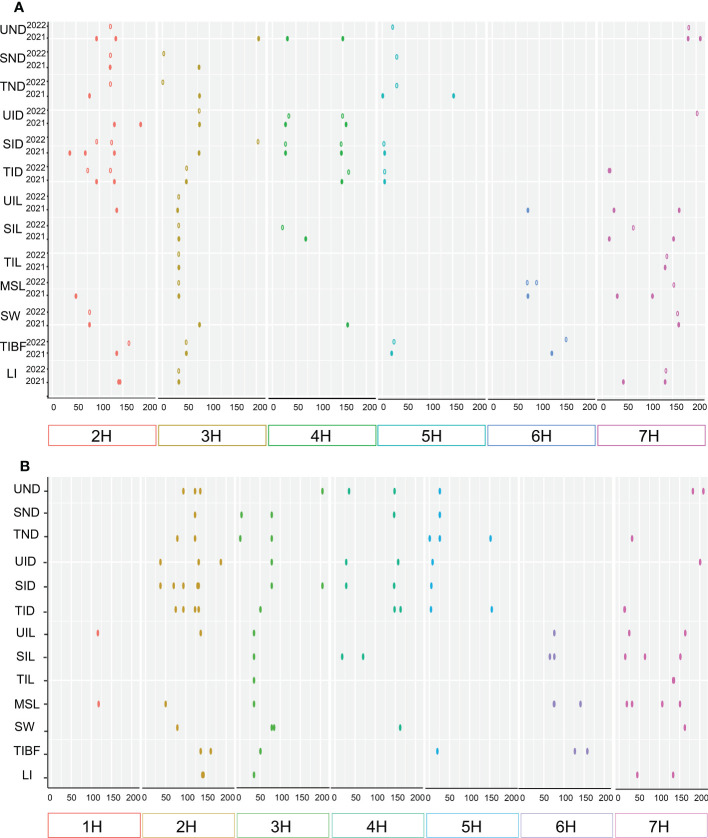
Chromosome distribution of QTLs associated with 13 stem related traits identified. **(A)** single-environment QTL analysis and **(B)** multi-environment trials (MET) analysis.

In total 103 QTLs were mapped on all chromosomes of barley except 1H for stem related traits using the ICIM BIP module in 2021 and 2022, including UND (10 QTLS), SND (5), TND (7), UID (9), SID (13), TID (12), UIL (6), SIL (7), TIL (4), MSL (9), SW (6), TIBF (8) and LI (7) (**Supplementary Table S1** and **Figure 3A**). Of them, the phenotypic variance of a single QTL was between 2.23% and 80.92%, with LOD values ranging from 3.01 to 32.68, 55(53.40%) major QTLs with PVE values greater than 10% were identified for UND (2 QTLS), SND (2), TND (3), UID (5), SID (6), TID (7), UIL (3), SIL (3), TIL (4), MSL (5), SW (4), TIBF (5) and LI (6) (**Supplementary Table S1**). 23 stable QTLs were detected for two consecutive years (**Table 2**), and 14 stable QTLs had PVE of more than 10% (two years average). Moreover, we identified 17 tightly linked or pleiotropic QTLs that influenced two or more traits, such as the QTL at 662.45-671.63 Mb on chromosome 2H simultaneously affected UIL, UND, UID, SID, TID and TIBF (**Supplementary Table S2**). To avoid the influence of row type (Rt), we used Rt as covariates for QTL mapping, a total of 91 QTLs were mapped, including 43 novel QTLs and 4 novel stable QTLs (**Supplementary Table S3** and **Table 2**).

**Table 2:** Major and stable QTLs identified for thirteen stem related traits in two years using multiple mapping method.

QTL	Chr.^a^	Position (cM)	Physical interval (Mb)	LOD	PVE^b^	ADD^c^	Year	Mapping method^d^
*qUND7-1*	7H	184	75.24-83.77	3.50-13.46	6.26-21.77	+	2021, 2022, BLUP	BIP, MET, Rt
*qSND2-1*	2H	123	645.22-645.37	7.34-10.11	16.11-18.99	+	2021, 2022, BLUP	BIP, MET
*qTND5-1*	5H	0/22.5	0.43-13.28	3.94-5.97	6.36-11.50	–	2021, 2022, BLUP	BIP, MET, Rt
*qUIL3-1*	3H	33	626.22-633.07	12.9-13.85	20.99-30.37	+	2021, 2022, BLUP	BIP, MET, Rt
*qSIL3-1*	3H	33	626.22-633.07	18.66-30.45	16.47-38.97	+	2021, 2022, BLUP	BIP, MET, Rt
*qTIL3-1*	3H	33	626.22-633.07	15.71-22.40	31.75-32.94	+	2021, 2022, BLUP	BIP, MET, Rt
*qTIL7-1*	7H	134/136	297.27-382.25	12.96-21.39	24.32-30.69	+	2021, 2022, BLUP	BIP, MET
*qUID3-1*	3H	79	530.99-532.66	6.85-6.98	12.67-15.73	–	2021, 2022, BLUP	BIP, MET, Rt
*qUID4-1*	4H	29	599.96-614.73	6.08-6.51	11.4-13.74	–	2021, 2022, BLUP	BIP, MET, Rt
*qUID4-2*	4H	151/158	7.86-16.17	4.20-4.68	7.01-10.44	+	2021, 2022, BLUP	BIP, MET, Rt
*qSID4-1*	4H	28	614.17-614.73	3.70-4.56	5.91-5.98	–	2021, 2022, BLUP	BIP, MET, Rt
*qSID4-2*	4H	148	15.50-16.98	10.52-10.54	15.56-19.65	+	2021, 2022, BLUP	BIP, MET, Rt
*qSID5-1*	5H	4	0.43-4.15	3.05-5.75	4.87-7.73	–	2021, 2022, BLUP	BIP, MET, Rt
*qTID3-1*	3H	52	602.92-603.14	4.42-8.82	5.58-12.76	–	2021, 2022	BIP, MET
*qTID5-1*	5H	4	0.43-4.15	5.05-8.82	6.52-12.82	–	2021, 2022, BLUP	BIP, MET, Rt
*qTIBF3-1*	3H	52	602.92-603.14	3.82-4.89	9.5-10.39	–	2021, 2022, BLUP	BIP, MET
*qTIBF5-1*	5H	17	6.81-9.87	4.68-5.19	10.31-15.48	–	2021, 2022, BLUP	BIP, MET
*qMSL3-1*	3H	33	626.22-633.07	17.02-26.35	17.52-39.85	+	2021, 2022, BLUP	BIP, MET, Rt
*qMSL6-1*	6H	77	510.42-517.96	5.68-10.90	4.58-11.72	+	2021, 2022, BLUP	BIP, MET, Rt
*qSW2-1*	2H	79	460.97-473.73	3.78-5.77	6.54-10.33	+	2021, 2022, BLUP	BIP, MET, Rt
*qSW7-1*	7H	160	167.59-214.88	6.34-9.60	11.40-19.27	+	2021, 2022, BLUP	BIP, MET, Rt
*qLI3-1*	3H	33	626.22-633.07	9.5-14.39	15.46-23.97	+	2021, 2022, BLUP	BIP, MET, Rt
*qLI7-2*	7H	134	297.27-382.25	12.98-18.06	22.69-32.56	+	2021, 2022, BLUP	BIP, MET
*qcUIL6-1*	6H	84	91.14-91.15	3.35-3.64	5.82-6.63	+	2021, 2022	Rt
*qcSIL7-1*	7H	137	337.03-358.83	10.07-21	14.65-43.57	+	2021, 2022	Rt
*qcUID3-2*	3H	220	12.78-23.92	3.17-3.22	6.18-9.17	–	2021, 2022	Rt
*qcMSL7-2*	7H	126	264.8-264.81	16.48-20.53	21.08-33.61	+	2021, 2022	Rt

^a^Chr, Chromosome; ^b^PVE, The phenotypic variation explained (in %) by each QTL; ^c^ADD, additive effect, positive values indicate that the alleles coming from Huadamai 6, negative values indicated that the alleles coming from Huaai 11; ^d^Mapping method, BIP (single-environment QTL analysis), MET (multi-environment trials analysis) and Rt (covariate QTL analysis).

In a multi-environment QTL analysis, 93 MET QTLs were identified for UND (9 QTLS), SND (5), TND (8), UID (8), SID (10), TID (10), UIL (6), SIL (8), TIL (3), MSL (10), SW (5), TIBF (6) and LI (5) (**Figure 3B** and **Supplementary Table S4**). Of them, 22 (23.66%) had PVE more than 10%. Besides, we found 21 QTL loci that influenced two or more traits synchronously detected in the ICIM MET module (**Supplementary Table S5**).

Further, to suppress the potential effect of the environment on stem lodging traits, we used the BLUP of stem traits for QTL analysis in the ICIM BIP module. 73QTLs were identified for UND (8 QTLS), SND (6), TND (8), UID (7), SID (7), TID (7), UIL (2), SIL (7), TIL (2), MSL (8), SW (5), TIBF (4) and LI (2) (**Supplementary Table S6**). Of them, the LOD values ranged from 3.03 to 32.23, and PVE of 26 (35.62%) QTLs was greater than 10%.

**Figure 4:**
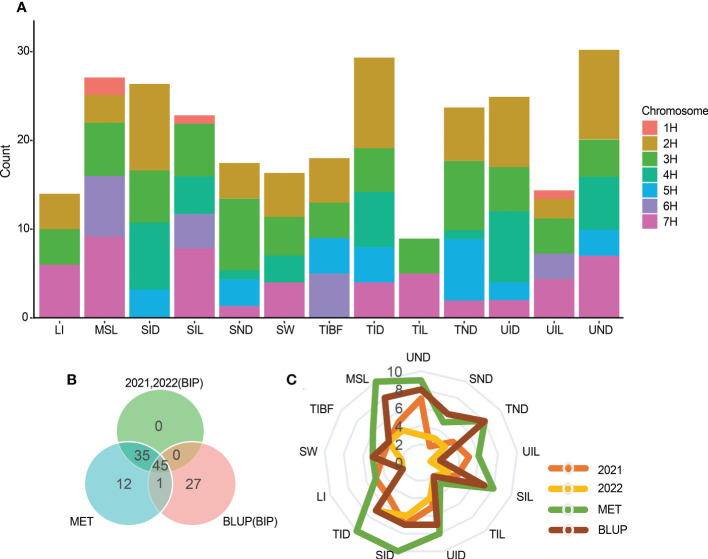
**(A)** Stacked map of QTL number of QTLs with 13 stem related traits identified on each chromosome. **(B)** Venn diagram of QTL detected by the three modes. **(C)** The number of QTL for a single trait in each year.

**Figure 5:**
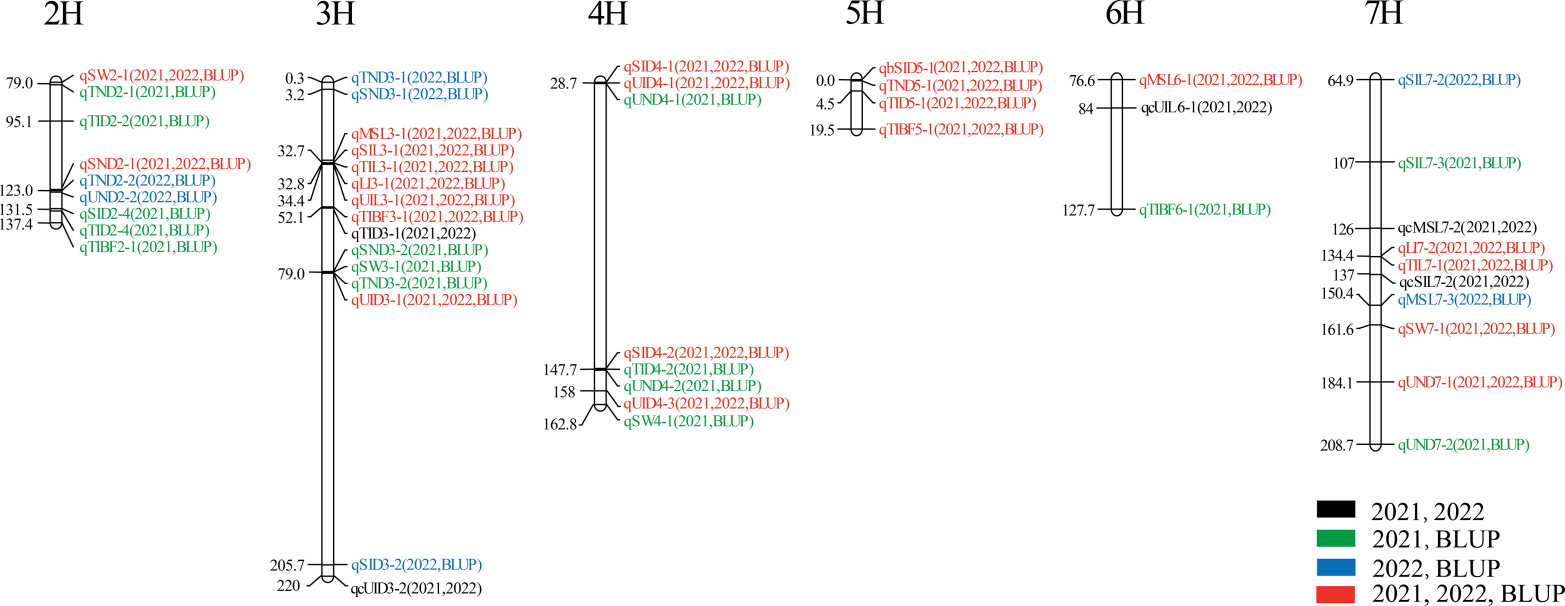
Chromosomes location of reliable QTLs for 13 stem traits.

Most QTLs detected by the three mapping methods were located on chromosomes 2H, 3H, and 7H (**Figure 4A**). **Figure 4B** is the Venn diagram of the QTL mapped in three mapping methods. 45 QTLs were mapped by all three mapping methods, and 36 QTLs were identified in the MET module and the BIP module. The number of QTL for a single trait in each year was shown in **Figure 4C**. Importantly, we identified 27 major and stable QTLs (QTLs of one trait repeatedly mapped in multiple mapping methods and in at least two years) or 49 stable QTLs (QTLs of one trait repeatedly mapped in at least one year and BLUP) (**Table 2** and **Figure 5**). The PVE of 49 stably QTLs was 3.79- 43.57%, with a LOD value from 3.01 to 32.68 (**Supplementary Table S7**). In addition, we integrated a large number of interlocking intervals for different traits. 22 regions were found on all chromosomes involving 288 QTLs, including 1, 6, 6, 3, 2, 1 and 3 intervals for chromosomes 1H, 2H, 3H, 4H, 5H, 6H and 7H, respectively (**Table 3**).

**Table 3:** Putative pleiotropy or linkage of QTLs on linkage groups in barley and other traits reported to be associated with these regions from the literatures.

Chr.^a^	Position (cM)	Physical interval (Mb)	Nur.^b^	Involed traits	Mapping method	Previous QTLs^c^
1H	118	29.73-36.73	3	UIL, MSL	MET, BLUP^d^	drought stress^21,22^; development^1^; malting quality^2^; biomass yield^3^; root traits^12^; water deficit^15^; flag leaf^18^
2H	38	182.8-185.87	3	UID, SID	BIP, MET	
2H	75/79	460.96-473.73	16	SID, TID, TND, TIL, SW	BIP, MET, BLUP, Rt	culm internode length^19^; drought^16,22^; development^1^; leaf blade area^7^; grain and spike number^8^; root Cl– content^9^; malting quality^2^; TKW, protein content^6^; seedling root traits^20^
2H	94/95	541.63-548.62	8	UND, SID, TID	BIP, MET, BLUP, Rt	
2H	123/129	645.22-651.53	15	SID, TID, UND, SND, TND, MSL	BIP, MET, BLUP	drought tolerance^20^; culm internode length^19^; seedling characteristics^24^; grain size and weight^25^; grain yield and phosphorus efficiency^14^; water deficit^15^; leaf length and area^23^; grain filling; flag leaf^18^
2H	132/137	662.45-671.63	14	UID, SID, TID, UND, UIL, TIBF	BIP, MET, BLUP	seedling characteristics^24^; leaf blade area^7^; biomass DSI, osmotic adjustment ^3^; seedling root traits^11,12^; leaf length salt stress^12^; water deficit^15,22^; grain filling^26^
2H	162	695.79-696.24	4	UID, TID, TIBF	BIP, MET, BLUP, Rt	grain size and weight^25^
3H	0/3	686.48-695.72	8	SND, TND	BIP, MET, BLUP, Rt	drought stress^21^
3H	33	626.22-633.07	31	TID, UIL, SIL, TIL, LI, MSL	BIP, MET, BLUP, Rt	drought stress^21^; seedling characteristics^24^; grain size and weight^25^; leaf length and area^23^; grain filling^26^
3H	52	599.15-602.92	7	TID, TIBF	BIP, MET, BLUP	grain size and weight^25^
3H	79	530.99-532.66	23	UID, SID, TID, UND, SND, TND, SIL, SW	BIP, MET, BLUP, Rt	seedling characteristics^24^; water deficit^15^
3H	205	34.96-37.47	12	SID, TID, UND, SND, TND	BIP, MET, BLUP, Rt	
3H	222	12.78-23.92	3	UID, SND	Rt	
4H	28	599.96-614.73	16	UID, SID, TID, UND	BIP, MET, BLUP, Rt	drought tolerance^20^
4H	148	14.61-22.49	23	UID, SID, TID, UND, SND, TND	BIP, MET, BLUP, Rt	leaf length and area^23^
4H	158/164	0.41-8.31	7	UID, TID, SW	BIP, MET, BLUP	leaf length and area^23^
5H	4/22	0.43-13.28	30	UID, SID, TID, UND, SND, TND, TIBF	BIP, MET, BLUP, Rt	grain size and weight^25^; water deficit^15^; grain filling^26^; flag leaf^18^; drought tolerance^20^, Drought Stress^15^
5H	153	379.45-428.15	4	TID, TND	BIP, MET, Rt	
6H	77	510.42-517.07	11	TID, UIL, SIL, MSL	BIP, MET, BLUP, Rt	lodging resistance^17^
7H	16/32	620.49-639.84	14	TID, TND, UIL, SIL, MSL	BIP, MET, BLUP, Rt	straw yield^10^; development^1^; tiller number^4^; leaf blade area^7^; seedling root traits^11^; leaf number in salt stress^13;^ water deficit^15^;drought^16,22^
7H	107/134/151	225.25-382.25	26	TID, SND, UIL, SIL, TIL, MSL, LI	BIP, MET, BLUP, Rt	grain size and weight^25^,grain filling^26^, flag leaf^18^
7H	160	166.04-214.88	10	TID, SND, TND, UIL, SW	BIP, MET, BLUP, Rt	drought stress^21^; grain size and weight^25^; grain yield and phosphorus efficiency^14^

^a^Chr, Chromosome; ^b^Nur, The number of QTLs included; ^c^Previous QTLs or Hotspot: ^1^ Alqudah et al. (2014), ^2^ Mohammadi et al. (2015), ^3^ Wehner et al. (2015), ^4^ Alqudah et al. (2016), ^5^ Maurer et al. (2016), ^6^ Pasam et al. (2012), ^7^ Alqudah et al. (2018), ^8^ Gawenda et al. (2015), ^9^ Long et al. (2013), ^10^ Al-Abdallat et al. (2017), ^11^ Abdel-Ghani et al. (2019), ^12^ Jia et al. (2019), ^13^ Ward et al. (2019), ^14^ Gong et al. (2016), ^15^ Moualeu-Ngangue et al. (2020), ^16^ Jabbari et al. (2018), ^17^ Kristensen et al. (2016), ^18^ Jabbari et al. (2019), ^19^ Sameri et al. (2009), ^20^ Tarawneh et al. (2020), ^21^ Zhang et al. (2019), ^22^ Dhanagond et al. (2019), ^23^ Du et al. (2019a), ^24^ Wang et al. (2017), ^25^ Wang et al. (2019), ^26^ Du et al. (2019b). ^d^BLUP : QTL analysis for BLUP was performed in BIP module.

### Genes located within major QTLs intervals

In total, 49 stably QTLs were detected, and 26 stable QTLs had PVE of more than 10% (**Supplementary Table S7**). We explored a large number of interlocking intervals for different traits (**Table 3**), such as the QTL at 662.45-671.63 Mb on chromosomal 2H simultaneously affected UIL, UND, UID, SID, TID and TIBF. We reviewed annotated information of the markers and identified potential candidate genes (**Supplementary Table S8**), based on the barley physical map (Cantalapiedra et al., 2015). 44 genes were located at 460.97-473.73 Mb interval on chromosome 2H. A total of 147 genes were located at 626.22-633.07Mb and 530.99-532.66Mb on chromosome 3H. And 244 genes were found in the interval (599.96-614.73 Mb) on chromosome 4H. QTLs located at approximately 6.81-9.87 Mb on chromosome 5H contained 101 genes. The important intervals on chromosome 7H were 81.89-83.77Mb and 297.27-382.25 Mb, containing 271 genes. We also looked for genes in several other interlocking loci, which included 360, 273, 383, 203, 160, and 794 genes selected for chromosomes 2H, 3H, 4H, 5H, 6H and 7H, respectively.

## Discussion

### Major and linkage QTLs for stem traits

Most of the studies on lodging focused on plant height and stem chemical composition (JSameri et al., 2009; Ren et al., 2013; Ren et al., 2014; Wehner et al., 2015; Gong et al., 2016; Wendt et al., 2016; Al-Abdallat et al., 2017; Bélanger et al., 2018; Hu et al., 2018; Pu et al., 2021), on heading date focused on the change of agronomic traits under drought stress and panicle traits in barley (Pasam et al., 2012; Long et al., 2013; Alqudah et al., 2014; Gawenda et al., 2015; Mohammadi et al., 2015; Alqudah et al., 2016; Kristensen et al., 2016; Maurer et al., 2016; Alqudah et al., 2018; Jabbari et al., 2018; Abdel-Ghani et al., 2019; Jia et al., 2019; Ward et al., 2019; Jabbari et al., 2019; Moualeu-Ngangue et al., 2020), but few studies on QTL mapping of stem diameter of internode or node were reported.

In our study, 27 stable and major QTLs were identified for stem related traits (**Table 2**). To notarize whether the QTLs here are new loci, we compared the physical positions with those stem related loci reported previously, and found some QTLs for stem traits were consistent with QTLs for seedling traits or grain traits mapped in previous reports (**Table 3**). For instance, different traits were mapped previously in the interval of 122-129 cM and 131-137 cM on chromosome 2H (Wang et al., 2017; Du et al., 2019a; Wang et al., 2019; Du et al., 2019b). Our research also showed evidence to support the possible pleiotropy of the *Vrs1* gene (Wang et al., 2016). Similarly, a large number of QTLs related to plant height related traits were also detected in the 626.22-633.07 Mb of chromosome 3H. In the same DH population, QTL for three internode length (Qith3-13) and heading date (Qhd3-13), were also near this region (Ren et al., 2013; Ren et al., 2014; Hu et al., 2018). In addition, in another hotspot on chromosome 5H at 0.43-13.28 Mb, the QTL of stem related traits we mapped was consistent with previously reported traits of grain filling rate or panicle related traits (Du et al., 2019b). These results showed certain correlation between barley stem and grain yield.

As expected, TIBF loci were mostly consistent with stem diameter loci, while LI loci were mostly consistent with internode length loci. In barley breeding, the relationship between traits should be considered, dwarf barley has lower grain yield due to its smaller biomass. We found that several interlocking intervals were simultaneously located by TID, TND, SND, MSL and LI (**Table 3**), which may be helpful to further explore the relationship between stem strength and plant height. QTL clusters of barley was iteratively reported (Qu et al., 2008; Schmalenbach and Pillen, 2009; Liu et al., 2015; Wang et al., 2016). The genetic mechanism of this general phenomenon might be gene linkage and pleiotropic effects in the same genomic region (Peng et al., 2003; Wang et al., 2015). Nevertheless, both linkage and pleiotropy need to be verified by further studies using cloning and fine mapping of gene or QTL.

### Possible genes associated with barley stem development

Many genes have been shown to be pleiotropic in barley. Such as sdw1/denso controls grain size, grain yield, the number of tillers, and plant height (Kuczyńska et al., 2014). Annotating these genes in each stable QTL interval by KEGG database, we found some of them were related to cell division and plant development (**Table 4**). The three genes detected in the chromosome 6H interval at 510.42-517.07 Mb might involve in the stem length of barley. *GS1a* gene (HORVU6Hr1G074030) plays a role in nitrogen sensing or signaling and the efficiency of photosynthetic or water use (Molina-Rueda et al., 2013). *RDRP1* gene (HORVU6Hr1G074220) and *RDRP2* gene (HORVU6Hr1G074180) regulate organic acid and amino acid metabolites, biogenesis and spikelet development by small RNA (Song et al., 2012; Jha et al., 2021). Another significant QTL area underlying traits of UIL, SIL, TIL, MSL, LI on chromosome 3H at 626.22-633.07 Mb was physically close to *HvGA20ox3* (HORVU3Hr1G089980) that encodes enzyme involved in gibberellin (GA) biosynthesis (Wendt et al., 2016; Bélanger et al., 2018). In addition, a region related to plant height traits was also detected on chromosome 7H at 297.27-382.25 Mb, the *14-3-3D* gene (HORVU7Hr1G061920) and *BRI1* gene (HORVU7Hr1G068990) located in the region plays an important intermediate in GA signal transduction and involves in the signaling of BRs (Pu et al., 2021), respectively. A stable QTL was detected near *HvVRT-2* (a flowering repressor regulated by vernalization and photoperiod) on chromosome 7H (81.89-83.77 Mb), the gene maintains the transition from vegetative stage to reproductive stage, thereby influencing spikelet morphology and the internode development (Kane et al., 2005; Szűcs et al., 2006).

**Table 4:** Twenty-three candidate genes from the target interval.

Candidate genes	Physical interval (Mb)	Involved traits	Potential function
HORVU2Hr1G066890	452.88-473.73	SW, UID, SID, TID, TND	DLAT (pyruvate dehydrogenase E2 component)
HORVU2Hr1G092760	645.22-651.53	MSL, UND, SND, TND, UID, SID, TID, TIBF	YUCCA (indole-3-pyruvate monooxygenase)
HORVU2Hr1G092290	651.53-653.98	MSL, UND, SND, TND, UID, SID, TID, TIBF	Vrs1/Int-d
HORVU2Hr1G093020	662.45-671.63	UIL, UND, TND, UID, SID, TID, TIBF	bglX (beta-glucosidase)
HORVU2Hr1G094090	662.45-671.63	UIL, UND, TND, UID, SID, TID, TIBF	KCS (3-ketoacyl-CoA synthase)
HORVU3Hr1G089450	626.22-633.07	TID, UIL, SIL, TIL, MSL, LI	SRP54 (signal recognition particle subunit)
HORVU3Hr1G089980	626.22-633.07	TID, UIL, SIL, TIL, MSL, LI	HvGA20ox3
HORVU4Hr1G006310	7.86-16.17	UND, SND, TND, UID, SID, TID, SW	DLAT (pyruvate dehydrogenase E2 component)
HORVU4Hr1G006070	7.86-16.17	UND, SND, TND, UID, SID, TID, SW	bglB (beta-glucosidase)
HORVU4Hr1G076460	599.96-614.73	UND, UID, SID, TID	HvRBOH
HORVU4Hr1G076250	599.96-614.73	UND, UID, SID, TID	BR-signaling kinase
HORVU5Hr1G006350	0.43-13.28	UND, SND, TND, UID, SID, TID, TIBF, SW	RHM (UDP-glucose 4,6-dehydratase)
HORVU6Hr1G074030	513.37-517.07	UIL, SIL, MSL	GS1a
HORVU6Hr1G074220	513.37-517.07	UIL, SIL, MSL	RDRP1
HORVU6Hr1G074180	513.37-517.07	UIL, SIL, MSL	RDRP2
HORVU7Hr1G036130	81.89-83.77	UND	HvVRT-2
HORVU7Hr1G049340	166.75-214.88	SW, UIL, SND, TND	DLAT (pyruvate dehydrogenase E2 component)
HORVU7Hr1G061920	288.27-382.25	SIL, TIL, MSL, LI	14-3-3D;
HORVU7Hr1G062120	288.27-382.25	SIL, TIL, MSL, LI	RBM25 (RNA-binding protein 25)
HORVU7Hr1G068990	288.27-382.25	SIL, TIL, MSL, LI	BRI1
HORVU7Hr1G108580	620.49-632.89	UIL, TND, MSL	DLAT (pyruvate dehydrogenase E2 component)
HORVU7Hr1G111190	620.49-632.89	UIL, TND, MSL	GBE (1,4-alpha-glucan branching enzyme)

Comparative genomics has shown that the functions of homologs are generally conserved. In addition to the partially functional identification of genes in barley, we found several potential candidate genes within the markers (**Table 4**), that might play a key role in stem development. HORVU2Hr1G066890, HORVU4Hr1G006310, HORVU7Hr1G108580 and HORVU7Hr1G049340 annotate pyruvate dehydrogenase E2 component (Bohne et al., 2013), might coordinate the synthesis of lipids and proteins for the biogenesis of photosynthetic membranes. The gene *SRP45* plays a role in chloroplast development in rice which is orthologs of the HORVU3Hr1G089450 (Zhang et al., 2013). HORVU2Hr1G093020 and HORVU4Hr1G006070 annotate beta-glucosidase, which is an important part of cellulase involved in various physiological processes in plants (Kawasaki et al., 2001). The GBE, a key enzyme in the catalytic regulation of α (1-6) glycosidic bond branch synthesis (Guan et al., 1995), annotated by HORVU7Hr1G111190. The gene (HORVU5Hr1G006350) that annotated as *RHM* (Saffer and Irish, 2018; Jiang et al., 2021), might be related to cell wall components. In addition, we identified several homologous genes associated with drought stress that might play a role in tiller and stem development. HORVU7Hr1G062120 annotates *RBM25*, an RNA binding protein in *Arabidopsis thaliana* that plays an important role in ABA-mediated alternative splicing and stress response (Cheng et al., 2017). YUCCA encodes a key auxin synthesis enzyme during drought stress of cotton (Wang et al., 2021), which is annotated by the HORVU7Hr1G067200. Another drought stress gene *KCS* encoded by HORVU2Hr1G094090 enhanced drought tolerance, increased the amount of chloroplast matrix, and increased stem diameter, stem coat thickness, growth rate, and lignin content in jute (Zhang et al., 2019; Tong et al., 2021). Moreover, two potential candidate genes (HORVU4Hr1G076460 and HORVU4Hr1G076250) were found next to each other on 4H, HORVU4Hr1G076460 encodes RBOH, DNA methylation of genes related to seed development affected by heat stress during grain filling (Mahalingam et al., 2021; Sakai et al., 2022). Brassinosteroid signaling kinases (BSK) encoded by HORVU4Hr1G076250 is a key family of receptor-like cytoplasmic kinases for BR signaling (Li et al., 2022), which is crucial for the development of plants, immunity and abiotic stress response, but these genes still are needed further verification in barley. Our studies showed that candidate genes may be involved in stem development and provide clues for further fine positioning.

### Stem traits for barley breeding programs

In this study, we measured the breaking force of the third internode and calculated the lodging index to observe the relationship between the traits after 28 days of heading (**Figure 2**). The results showed that TIBF was negatively correlated with LI and TIL. Compared with the strength trait, the correlation between LI and plant height trait was highly significant. All the traits were significant and positively correlated with SW. And the diameter of each stem node was positively correlated with the stem diameter of each internode. SIL and TIL had no correlation with SID and TID, a similar phenomenon occurs in rice (Guo et al., 2021), suggesting that improving stem strength might not change plant height.

Stem strength can be affected by various factors. The leaf sheath could provide great physical support (Cornwall et al., 2021). Lodging resistance is usually related to stem diameter. Simonneau et al. (1993) showed that during drought stress stem diameters contract in response to changes in internal water status. Therefore, it can be used as a selection criterion for stress tolerance (Sallam et al., 2015). In our study, the finding is reflected in the consistency of QTL loci. Then, the study of the genetic basis of stem development may help us to fight global climate change.

Collectively, because of the importance of plant height and stem diameter in lodging resistance, stress resistance, grain yield, and photosynthate transport in barley, a stem with a suitable height and strength is usually preferred in barley breeding.

## Conclusions

In this study, 27 stable and major QTLs, and 22 QTL clustered regions were identified for 13 stem related traits. We also methodically compared the genetic correspondence with other different traits at the same locus. In total, 22 genes were identified as promising candidates associated with plant development and adverse stress, which were closely related to lodging resistance, stress resistance, photosynthate transport and grain development in barley. The identification of QTL conferring stem related traits can help us to know the genetic basis of stem and ameliorate lodging resistance potential of barley, and is useful in future marker-assisted barley breeding programs.

## Data availability statement

The original contributions presented in the study are included in the article/**Supplementary Material**. Further inquiries can be directed to the corresponding author.

## Author contributions

XR and AZ conceived the study and worked on the approval of the manuscript. AZ, XH, TZ, HP and YA performed the experiments. AZ wrote the first draft. DS, GS, CL and XR revised the manuscript. AZ and YZ contributed to data analysis and managed reagents. All authors contributed to the article and approved the submitted version.

## Funding

This research was supported by the earmarked fund for China Agriculture Research System (CARS-5).

## Conflict of interest

The authors declare that the research was conducted in the absence of any commercial or financial relationships that could be construed as a potential conflict of interest.

## Publisher’s note

All claims expressed in this article are solely those of the authors and do not necessarily represent those of their affiliated organizations, or those of the publisher, the editors and the reviewers. Any product that may be evaluated in this article, or claim that may be made by its manufacturer, is not guaranteed or endorsed by the publisher.
