# Supplementary material for: Identification of QTL underlying the main stem related traits in a doubled haploid barley population
Source: Front Plant Sci. 2022 Dec 1;13:1063988. doi: 10.3389/fpls.2022.1063988 (PMC9751491; doi:10.3389/fpls.2022.1063988)
Supplement: Supplementary file 1 [file Table_1.doc]

Figure S1. Measurement of the third internode breaking force (TIBF)
